# Breaking the symmetry of nanosphere lithography with anisotropic plasma etching induced by temperature gradients†

**DOI:** 10.1039/d0na00718h

**Published:** 2020-12-11

**Authors:** Daniel Darvill, Marzia Iarossi, Ricardo M. Abraham Ekeroth, Aliaksandr Hubarevich, Jian-An Huang, Francesco De Angelis

**Affiliations:** Istituto Italiano di Tecnologia Via Morego 30 16136 Genova Italy Francesco.DeAngelis@iit.it; Dipartimento di Informatica, Bioingegneria, Robotica e Ingegneria dei Sistemi (DIBRIS), Università; degli Studi di Genova Via Balbi 5 16126 Genova Italy; Instituto de Física Arroyo Seco (CIFICEN-CICPBA-CONICET), Universidad Nacional del Centro de la Provincia de Buenos Aires Pinto 399 7000 Tandil Argentina

## Abstract

We report a novel anisotropic process, termed plasma etching induced by temperature gradients (PE-TG), which we use to modify the 3D morphology of a hexagonally close-packed polystyrene sphere array. Specifically, we combined an isotropic oxygen plasma (generated by a plasma cleaner) and a vertical temperature gradient applied from the bottom to the top of a colloidal mask to create an anisotropic etching process. As a result, an ordered array of well-defined and separated nano mushrooms is obtained. We demonstrate that the features of the mushrooms, namely the hat size and their intrinsic undercut, as well as the pillar diameter and height, can be easily tuned by adjusting the main parameters of the process *i.e.* the temperature gradient and etching time, or the spheres' size. We show that PS mushroom arrays can be used as nanostructured templates to fabricate plasmonic arrays, such as gold-capped nano mushrooms and ultra-small nanoapertures, by using vertical and oblique gold sputtering deposition respectively. PE-TG reveals a new, cheap and facile approach to produce plasmonic nanostructures of great interest in the fields of molecular sensing, surface-enhanced Raman scattering (SERS), energy harvesting and optoelectronics. We study the optical properties of the Au-capped nano mushroom arrays and their performance as biosensing platforms by performing SERS measurements.

## Abstract 2

A strongly anisotropic etching process mediated by temperature is presented to create nano mushroom arrays from a monolayer of spheres.

## Graphical abstract

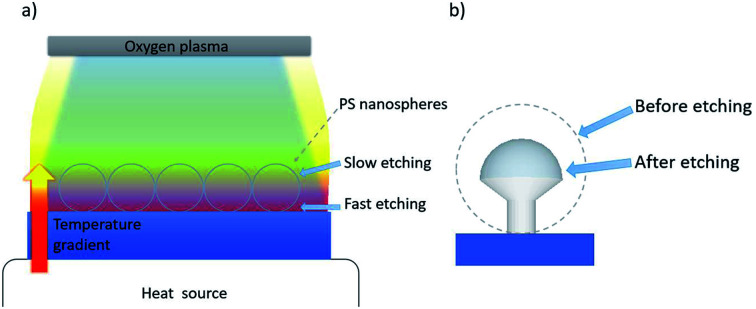

## Introduction

In the past decades, self-assembly of colloidal beads, such as polystyrene (PS) and silicon dioxide spheres, has been widely investigated for the large-scale fabrication of periodic micro- and nanostructure arrays.^1–3^ Highly monodispersed colloidal spheres with sizes spanning from tens of nanometers up to tens of micrometers are commercially available or can be easily prepared by emulsion polymerization and sol–gel technique.^4^ Due to their high monodispersity in size and shape, colloidal spheres can be self-assembled into close-packed 2D monolayers^5^ and 3D periodic multilayer arrays by using dip coating,^6,7^ interface assembly-techniques,^8–10^ electrophoretic deposition^11^ and spin coating.^12,13^ Such arrays are then used as templates or masks for surface patterning^14,15^ establishing a powerful and popular nanofabrication technique with low cost, high throughput and good reproducibility,^16,17^ referred to as colloidal lithography or nanosphere lithography (NSL).^18^

Other processes, such as etching,^19,20^ deposition^21^ and surface decoration^22,23^ are usually performed on colloidal masks to generate functional periodic nanostructures with optical,^24–26^ magnetic,^27,28^ plasmonic^29–31^ or catalytic^32,33^ properties. Amongst other applications, plasmonic nano arrays,^34–38^ made of metal coated arrays of nanospheres obtained from bottom-up approaches, have been widely employed as effective and low-cost sensing platforms^39^ for surface-enhanced Raman spectroscopy (SERS) due to their electromagnetic field enhancement,^40^ known as hot-spots, originating from gap modes, tip modes and nanocavity modes.^41–45^ These enhancement mechanisms provide a way to enhance the weak scattering signal of the molecules of interest, which in turn represent their molecular fingerprint.^46–48^ Throughout the years, many etching processes and their parameters have been explored to further improve the capabilities of the masks. In particular, many efforts have been devoted to not only control their shape in-plane but also perpendicularly to alter their 3D structure. In this regard, hexagonally close-packed (hcp) monolayer arrays of PS colloidal spheres can be treated with reactive ion etching (RIE) or other oxygen plasma treatments^49,50^ to produce ordered non-close packed arrays. Adjusting the plasma etching parameters, the size of the spheres can be shrunk down to the desired dimension, whilst increasing the interparticle distance. This process keeps the position of each sphere in the hexagonal array unchanged, but can also increase the surface roughness due to polymer degradation and cross-linking.

During plasma etching processes carried out *via* RIE, PS spheres rapidly decrease in diameter and often undergo shape modification from spheres to oblate spheroids.^51^ Nevertheless, very accurate isotropic etching processes with low etching rates performed with RIE at low temperature (−150 °C) have been reported.^52^ Alternatively, the etching can be performed with a plasma cleaner to slowly shrink the particles and eventually modify their shape from spherical to polyhedral with hexagonal cross sections.^53^

Although NSL has been successfully used to produce various periodic micro- and nanostructures, colloidal masks are still limited due to the spherical shape of the particles. In fact, controlling “out of plane” geometry still represents one of the most common limitations of mask-based approaches. In addition, the manipulation of colloidal arrays to create 3D non-spherical structures often requires multiple and time-consuming fabrication steps, such as ion milling and etching of sacrificial layers. Nevertheless, several complex or asymmetric nanostructure designs have been already proposed.^54,55^ In fact, depositions of different materials, such as metals and metal oxides^56,57^ by vertical and oblique angle depositions have been successfully explored in both high/ultrahigh systems, such as electron beam and thermal evaporation, and low vacuum systems, such as direct current sputtering and chemical vapor deposition.^58–60^ However, these approaches are limited in scope as they are based on similar masks where the symmetry has not been overcome. Indeed, no one has explored temperature gradients as the key-enabling parameter to control mask shapes.

**Fig. 1:**
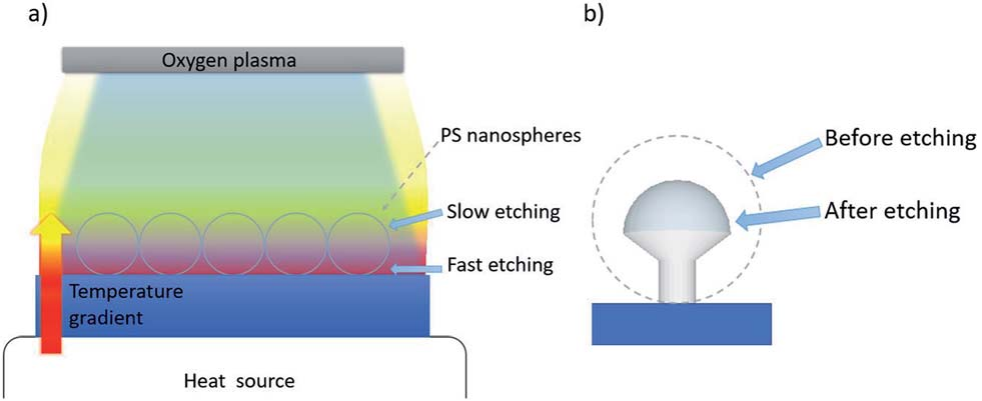
(a) Scheme of vertical temperature gradient applied together with an oxygen plasma to create an anisotropic etching process utilizing ordered PS nanospheres array. (b) Morphology of a PS nano mushroom after the etching process induced by a vertical temperature gradient.

Here, we report a new and cheap anisotropic plasma etching process that combines an isotropic oxygen plasma etching performed with a bench radio frequency plasma cleaner and a vertical temperature gradient applied from the bottom to the top of a hcp PS sphere monolayer (Fig. 1a). This process leads to a strong anisotropic etching along the *z*-direction, allowing us to control the morphology of the particles and shape them, into well-defined non-close packed nano mushroom arrays (Fig. 1b). A uniform change in temperature will only alter the etching rate but a temperature gradient introduces anisotropy across the structure varying the etching rate as a spatial function. As a result of heat accumulation, the etching rate is higher on the underside of the colloidal mask and thus the particles' hemisphere in contact with the substrate etches faster. This concept can be generalized to a branch of yet unexplored etching processes based on local temperature gradients that we have termed as plasma etching induced by temperature gradients (PE-TG). We demonstrate that the PE-TG process modifies the morphology of the spheres beyond the resolution of conventional NSL by using a temperature gradient as an additional parameter during etching processes both experimentally and theoretically. In addition, we show that the PS mushrooms produced can be used as a nanostructured template to easily produce various demanded plasmonic nanostructures, such as Au-capped nano mushroom arrays and ultra-small nanoapertures.^61–64^ Although we have concentrated our attention on colloidal masks of PS spheres, PE-TG can be potentially explored for the etching of other polymeric particles, or even core–shell particles with a polymeric shell allowing you, with a pre-etch of the polymer shell, to alter the interparticle distances,^65^ and in prospective overcome similar barriers encountered in other lithographic methods, for example optical mask lithographies, where creating patterns with an undercut is still challenging.^66,67^ Fabrication aspects aside, we investigate the optical properties of the Au-capped nano mushroom arrays and their effectiveness as SERS substrate for biosensing applications.

## Results and discussion

**Fig. 2:**
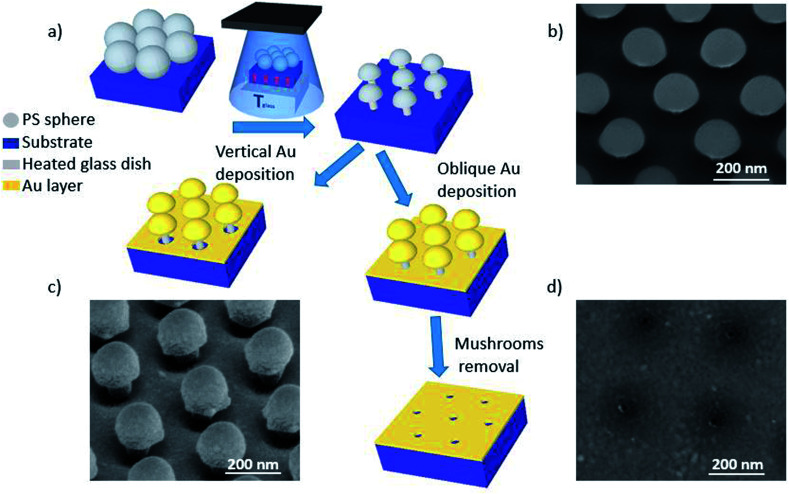
(a) A schematic flow chart of the fabrication process. Firstly, the colloidal mask is treated with PE-TG. The sample is in contact with a glass dish preheated in an oven to temperature *T*_glass_ to apply an external temperature gradient during the oxygen plasma exposure. As a result, a nanostructured colloidal mask of separated periodic nano mushrooms is obtained. Then, sputtering deposition of Au with one of two cathode/target orientations is performed: vertical or oblique. The principal difference is that after the vertical deposition an area around the pillar is not covered with Au whilst the space underneath the mushrooms' hat is completely filled after the oblique deposition. In the latter case, mushroom hats are removed by tape and an array of ultra-small nanoapertures are left on the substrate. (b) SEM image (tilted-view) of PS nano mushroom arrays obtained from a hcp monolayer of spheres with initial diameter of 300 nm after PE-TG process on silicon (*T*_glass_ = 65 °C, exposure time: 120 s, oxygen flow rate: 20 sccm). (c) SEM image (tilted-view) of the PS nano mushroom arrays after the vertical deposition of Au (30 nm) on glass. (d) SEM image of the nanoapertures obtained removing the nanostructures after the deposition of Au at an oblique cathode/target orientation on silicon.

The etching process we have developed is applied to hcp PS sphere arrays to shape in 3D their morphology. As schematically shown in Fig. 2a, a slow and isotropic oxygen etching is performed in a plasma cleaner chamber while the substrate is in contact with a glass dish heated to temperature *T*_glass_, higher than the room temperature (20 °C) and well-below the PS glass transition temperature (approximately 100 °C). The temperature difference between the bottom and top of the PS spheres causes a variation of the etching rate along the *z*-direction, *i.e.* the bottom hemisphere shrinks faster than the top one. This results in the bottom hemisphere becoming a pillar while the top half reduces in size without losing its hemispherical shape.

After the PE-TG process, the hcp PS sphere arrays are completely modified into well-separated mushroom array. An example SEM image of PS sphere arrays with initial diameter of 300 nm treated with PE-TG is reported in Fig. 2b. Remarkably, the PE-TG process allows one to realize a nanostructured colloidal mask where both the pillar and hat features of the mushrooms are controllable, making a simple fabrication route for the production of various plasmonic nanostructures. We have explored different Au deposition configurations to prove the versatility of the polystyrene nano mushrooms as a mask. A sputter coater with a vertical cathode/target orientation can be used to deposit Au on the sample while leaving uncovered an area around the bottom part of the pillars. As a result, periodic mushrooms with an Au cap separated from the Au film by holes are obtained, as shown from the SEM image in Fig. 2c. Alternatively, the whole space under the mushrooms hat is filled by performing the sputter deposition with a non-vertical orientation between cathode and target. Following removal of the mushrooms with tape, this provides a convenient strategy to create a pattern of small periodic nanoapertures by exploiting the size of the mushrooms' pillar (see an example in the SEM image in Fig. 2d).

**Fig. 3:**
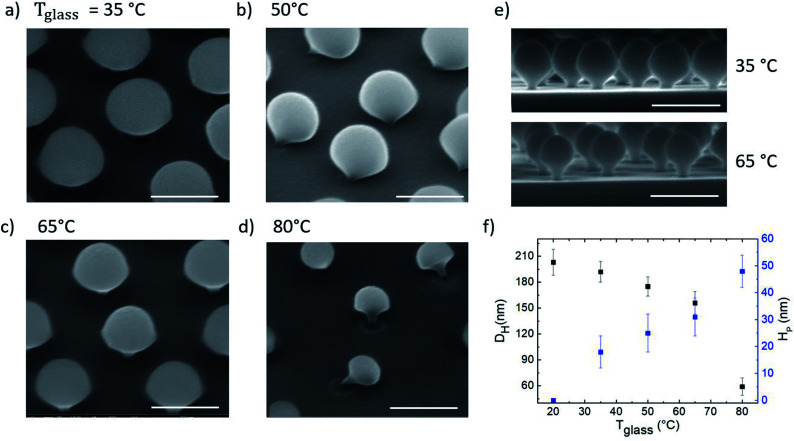
SEM images (tilted-view, scale bar: 200 nm) of hcp PS spheres on silicon treated with PE-TG for 120 s where (a) *T*_glass_ = 35 °C, (b) 50 °C, (c) 65 °C and (d) 80 °C respectively. Nano mushrooms in (b) and (d) are intentionally knocked over with long exposure to electron beam to show underside morphology. In (e) cross-section SEM images (scale bar: 300 nm) of mushrooms array from PE-TG process with *T*_glass_ = 35 °C and 65 °C, top and bottom respectively. In (f) mushrooms hat diameter *D*_H_ and pillar height *H*_P_ as a function of *T*_glass_.

In order to investigate how the vertical temperature gradient affects the etching process, four samples, consisting of arrays of hcp PS spheres (300 nm in diameter) on Si substrates, were exposed to the oxygen plasma treatment for 120 s while in contact with a glass dish at different temperatures from *T*_glass_ = 35 °C to 80 °C, with steps of 15 °C respectively. SEM images of these samples after the PE-TG treatment are shown in Fig. 3a–d. From the SEM images it is clear that the vertical temperature gradient shapes PS spheres into mushrooms. Their features, namely the hat size and the pillar diameter and height, can be tailored by changing the value of the applied temperature gradient. In fact, when the glass dish is at room temperature, *i.e.* no temperature gradient is experienced by the sample, the exposure to the oxygen plasma for 120 s reduces the particles diameter to an average value of 180 nm but they are still spherical (see Fig. S1†). Furthermore, we also found a linear relationship between the spheres' diameter and the etching time with an etching rate of about 1 nm s^−1^ (see Fig. S2 and S3†). If the temperature of the glass dish is higher than the room temperature, heat flow is accumulated at the bottom hemisphere of the hcp PS array and then dissipated to the top, thus increasing the etching rate on the bottom. At *T*_glass_ = 35 °C, slightly higher than room temperature, mushrooms were found with a hat size *D*_H_ of about 180 nm and a pillar height *H*_P_ of about 23 nm. Further increase of the temperature up to 80 °C modifies the mushrooms morphology to *D*_H_ = 130 nm and *H*_P_ = 50 nm. In Fig. 3e the cross-section SEM images of mushrooms obtained by using PE-TG at 35 °C and 65 °C are shown in order to highlight how their morphology changes as a function of the temperature. Increasing *T*_glass_ and keeping the etching time constant, both the hat and pillar sizes become smaller, while the pillar's height increases, as shown from the analysis reported in Fig. 3f. Here the process has been characterized on silicon but nano mushrooms can still be produced for other substrates such as glass, it is found due to the different thermal conductivities of the substrate that the shape of the mushrooms varies slightly as on glass the etching rate is slower and thus the pillars are slightly thicker and shorter. However, it is not possible to increase the temperature even more to avoid polymer residues and degradation occurring when the glass transition temperature of PS is approached. The nano mushrooms in Fig. 3b and d have been knocked over by long exposure to the SEM electron beam to better show the change in underside morphology for different temperature processes. Without this beam damage etching step the PS mushrooms are found to all remain upright and stable after metal deposition or drop casting of solutions onto the substrate.

It is already well known if the surface temperature of the material increases so does the etching rate during an etching process. The faster etching rate is found to be a combination of factors relating to the higher speed of the reacting species, neutral atom recombination and exothermic carbon oxidation.^68^ In our case as the surface temperature is not isotropic around the sphere, the etching rate also varies relative to the temperature distribution.

**Fig. 4:**
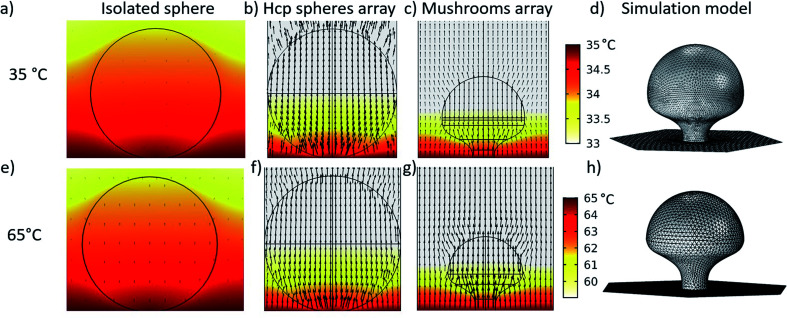
Thermodynamic simulations to explain the heat distribution inside the oxygen plasma chamber aiding the anisotropic etching during the PE-TG process. Simulations for *T*_glass_ = 35 °C and 65 °C divided between the top (a–d) and bottom row (e–h) respectively. PS spheres of 300 nm in diameter are simulated for an isolated sphere (a and e), sphere in hcp array (b and f) and PS mushroom shape in array after 120 s PE-TG process for first, second and third column respectively. The color maps show the temperature distributions and the black arrows represent the heat flow, both magnitudes correspond to the stationary regime. Color bars are relative to all simulations on that row and black arrows are scaled to 5 μW m^−2^ and 2.8 μW m^−2^ with a logarithmic spatial distribution for *T*_glass_ = 35 °C and 65 °C respectively. (d and h) Simulated geometries of PS mushroom shapes after 120 s PE-TG deduced from SEM images. Wigner–Seitz cells are used for the array simulations.

In order to understand how the temperature gradient applied during the PE-TG process leads to anisotropic etching, thermodynamic simulations of the system were conducted in Fig. 4. In our case, a heat source is applied to the bottom side of the substrate (*T*_glass_) creating a vertical temperature gradient across the PS spheres atop the substrate. The top row (Fig. 3a–d) refers to the case of *T*_glass_ = 35 °C whilst the bottom row (Fig. 4e and f) corresponds to *T*_glass_ = 65 °C. For both temperatures three different simulations are given; the first column (Fig. 4a and e) is for an isolated sphere, the second column (Fig. 4b and f) is for a single sphere in the hcp PS monolayer and the third column (Fig. 4c and g) is related to the final mushroom shape produced from the PS sphere at the end of the etching process. These colourmaps simulate a cross section of the temperature distribution around the particles, where the temperature bars are common to all the simulations on that row. The black arrows represent the magnitude of the stationary heat flow in each case. The final column (Fig. 4d and h) illustrates the simulated geometries of the final mushroom shape deduced from SEM images of samples fabricated by PE-TG. In all simulations the bottom plane defines the point of contact between the PS sphere and the heated substrate. The fluid dynamics of the surrounding air at pressure *P* inside the plasma cleaner was taken into account, as well as the heat radiation emitted by the substrate and the particles. Wigner–Seitz cells were used in the arrays' simulations to save computational time. Simulations were carried out in Comsol using the heat transfer and microfluidics modules coupled through the non-isothermal flow multiphysics interface. Further details of the simulations can be found in the ESI.†

By first considering the simulations for *T*_glass_ = 35 °C, it can be seen that for an isolated sphere of 300 nm in diameter (Fig. 4a) almost the whole sphere equilibrates in temperature to *T*_glass_ and the heat flow is low. Conversely, for the case of a sphere in an hcp monolayer (Fig. 4b), a temperature difference between the top and bottom half of the sphere is seen. Although there is not a large difference between *T*_glass_ and room temperature, a temperature difference of 2 °C is obtained vertically across the sphere. The accumulation of heat beneath the particles is due to the trapping of the heat radiated from the substrate by the hcp spheres array and is enough to induce anisotropic etching. It should be noted that the heat flow around the bottom of the sphere is directed upwards and towards the sphere due to the neighboring spheres. Thus, this heat flow contributes to enhance the etching rate at the bottom hemisphere of the spheres when they are ordered in hcp arrays, leading to the final mushroom shape after the exposure to the PE-TG process (Fig. 4d). As etching occurs, the heat still flows towards and through the pillar of the mushrooms altering the shape over time until the final structure is obtained. As there is still heat flow towards the bottom hemisphere this ensures a continuous controllable evolution of the mushroom shape over time (see Fig. 4c). These inferences are supported experimentally from applying PE-TG on a sample with both compact grains and isolated spheres. Only the former are found to create mushrooms whilst the latter remain spherical (Fig. S4†).

Another remarkable effect is that the distribution of the heat difference remains in the lower half of the PS even as the sphere shrinks in size from a sphere to a mushroom. As a consequence, mushrooms will start to fall with time as the pillar base will be shrunk due to the locally-high etching rate until it cannot support the mushroom hat.

A similar analysis was performed for *T*_glass_ = 65 °C, again taking an isolated sphere of 300 nm as the starting point (Fig. 4e). For the higher temperature simulations, almost no temperature difference towards an isolated sphere is observed. This result supports the experimental fact that no well-defined mushroom can be obtained from without the presence of adjacent spheres. Moving to the case of a PS sphere in the hcp array (Fig. 4f), a temperature difference up to 6 °C is obtained. Although the gradient is higher, the temperature difference is again concentrated across the bottom hemisphere as for the case of *T*_glass_ = 35 °C, which induces the necessary conditions for anisotropic etching to create the mushroom shape. The same inferences can also be made for the mushroom shape (Fig. 4g) where temperature differences are localized across the lower hemisphere and the heat flow acts towards and upwards through the mushroom shape as it takes form. As has already been noted experimentally, the shape for the final mushroom has a more noticeable undercut mushroom head and the pillar is thinner than the one treated at 35 °C.

It is worth noticing that the pillar is etched faster with a higher temperature and therefore the process etches the pillar to the same point of failure in a faster time than the lower temperature case. The fact that changing *T*_glass_ from 35 °C to 80 °C alters the profile of temperature difference inside the polystyrene sphere is shown in the ESI (Fig. S5†).

Leading on from a discussion of how the PE-TG process works, we can now discuss the various plasmonic nanostructures produced by applying this process to close-packed PS spheres. Previously, Au-capped mushroom arrays were fabricated with writing tools, such as two-beam interference lithography to pattern a periodic array of photoresist pillars in a square lattice followed by thermal evaporation.^61^ Another similarly shaped Au nanostructure, termed disk-coupled dots on pillar antenna array, was investigated and shows good results but their fabrication is based on nanoimprint lithography that requires complex techniques to create a periodic 2D pillar mold.^69^ A benefit of the PS mushroom arrays obtained with PE-TG is that their shape allows one to produce plasmonic nano mushrooms by simply depositing an Au layer with a vertical cathode/target orientation. It has already been shown that such Au mushroom arrays can be used for extraordinary light transmission,^70^ enhancement of Raman signals,^71–73^ high sensing performances based on fluorescence enhancement,^74^ and colour generation of large areas with plasmonic nanostructures.^75,76^

**Fig. 5:**
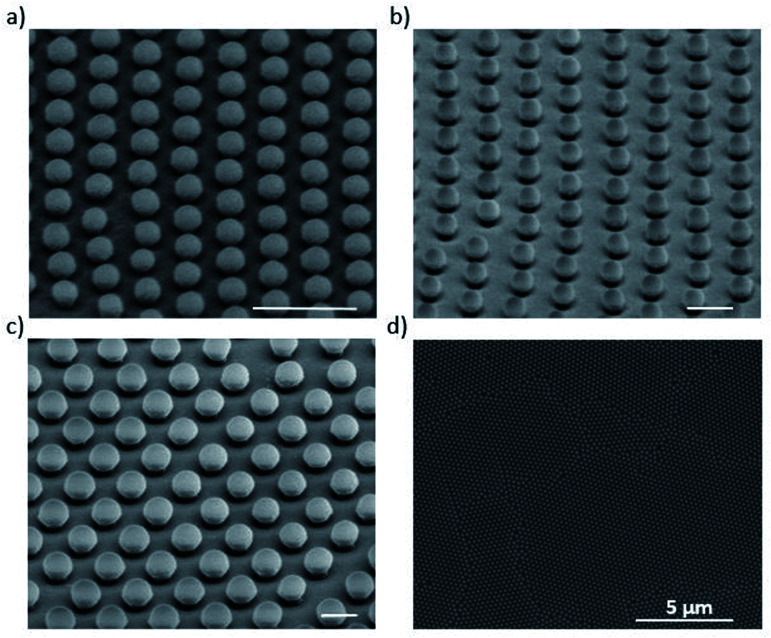
SEM images (tilted-view, scale bar: 300 nm) of Au capped nano mushrooms on silicon obtained from the treatment with PE-TG process at 65 °C of PS nanospheres arrays with different initial diameters: (a) 140 nm, (b) 252 nm and (c) 420 nm respectively. The thickness of the deposited Au layer is 20 nm. (d) SEM image of a large area of a sample to show the quality of the distribution of the nano mushrooms' array.

Before discussing the optical properties of the Au-capped mushroom arrays, it is important to note that even if the anisotropy of this etching process depends on both the applied temperature gradient and the presence of the ordered array to sustain the heat flow in the bottom hemisphere of the particles, it does not depend on a particular diameter of the PS spheres. In order to prove this point hcp arrays of PS spheres with different diameters of 140, 252 and 420 nm were treated with the PE-TG process at 65 °C and nano mushrooms of different sizes were found in all the cases. In Fig. 5a–c SEM images of the corresponding Au-capped mushroom arrays after the deposition of 20 nm of Au are reported (Au cap mean diameter of approximately 60, 150 and 260 nm respectively; gap value about of 13, 40 and 55 nm respectively). Here, the presence of a gap between the Au cap and the film is well-visible and, as explained below, plays an important role for their optical properties (see how the gap decreases by increasing the thickness of the deposited Au layer from the cross-section SEM images of the Au-capped nano mushrooms in Fig. S6†). However, such gaps between the Au film and the cap of the mushrooms is not a particular feature of mushrooms because it also exists for non-modified spherical templates after a metal deposition, as already reported in literature.^77,78^ From these SEM images it is also clear that the nano mushrooms all stand vertically and thus, the stability of the structures is not an issue and can be controlled by tuning the dimensions of the pillar compared to the size of the hat through the etching process and further improved by the deposition of the Au layer. Indeed, the quality of the distribution of the Au-capped nano mushrooms and their order is affected by the size of the grains of the hcp PS sphere arrays which in turns depends on the technique used to form the monolayer on the substrate. The SEM image in Fig. 5d shows the quality of the distribution of the Au-capped nano mushrooms by our chosen lithography method.

**Fig. 6:**
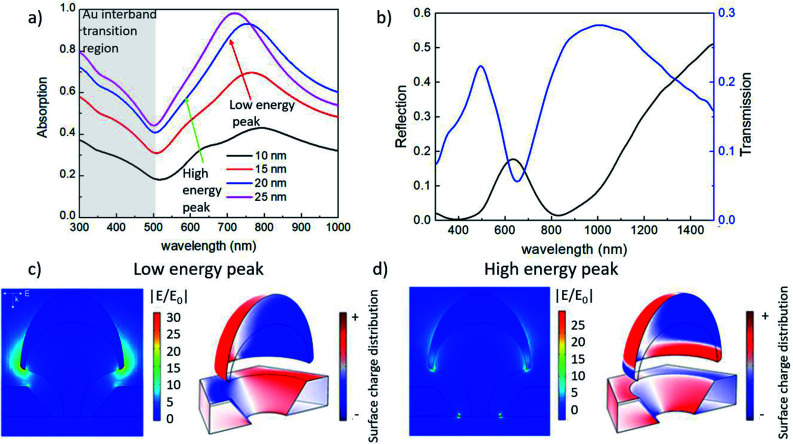
In (a) absorption spectra of Au nano mushrooms arrays on glass as a function of the Au thickness, respectively. In (b) transmittance and reflectance spectra of mushroom arrays covered with 25 nm of Au for p polarized light at 20° angle of incidence. In (c) and (d) simulated electric field and surface charge distributions of the low and high energy peaks, respectively.

In order to characterize the optical properties of such nanostructures, Au-capped nano mushrooms with a hat size of about 90 nm obtained from a hcp array of 140 nm spheres treated with the PE-TG process was covered with sequential depositions of Au. Doing so the gap size between the Au cap and the film was decreased each time. The deposited Au film was varied in thickness from 10 to 25 nm and, after each deposition, the corresponding absorption spectra was recorded, as shown in Fig. 6a. The absorption spectrum shows two peaks: the lower energy peak is related to the plasmonic resonance of the gold cap tip and Au film mode; while the higher energy peak is related to the plasmonic quadrupole resonance of the bulk cap and of the gold film hole (see the surface charge distribution in Fig. 6c and d, respectively).^79,80^ A different phase between the bottom part of the Au cap and top surface of the Au film, as well as the small gap spacing of 10–20 nm, creates a strong electric field enhancement for both peaks (see |*E*/*E*_0_| examples in Fig. 6c and d), which makes such structures a highly sensitive detector for biological applications. If the distance of the cap and film are carefully controlled it should be possible to couple the low energy modes creating a strong near field in the gap.^81^ After each deposition, both a blue-shift and an enhancement of the absorption have been observed. The blue shift is mainly due to the increase of the Au thickness around the mushroom hat.^82^ A stronger absorption and shift are seen for the 25 nm deposition, which can be attributed to the increased thickness of gold and how this affects the tip shape as well as reaching a limit for light that can transmit through the gap.^83–85^ The absorption below 500 nm is mainly related to the interband transition in Au.^86^ Furthermore, Fig. 6b shows the transmittance and reflectance measurements for p-polarized light at a 20° angle of incidence for Au-capped nano mushroom arrays of 150 nm after the deposition of 25 nm of Au. Compared to the transmission from nanohole arrays, the second peak of the transmission spectrum is enhanced as a result of the presence of the Au cap which acts as a blocker for the incident light, as elsewhere reported in literature.^71,87,88^ Samples were measured at multiple locations and the signal was found to be uniform across the substrates, further optical characterization is reported in the ESI† to show that the optical properties can be tailored by changing the dimensions of the nanostructures (Fig. S7 and S8†).

**Fig. 7:**
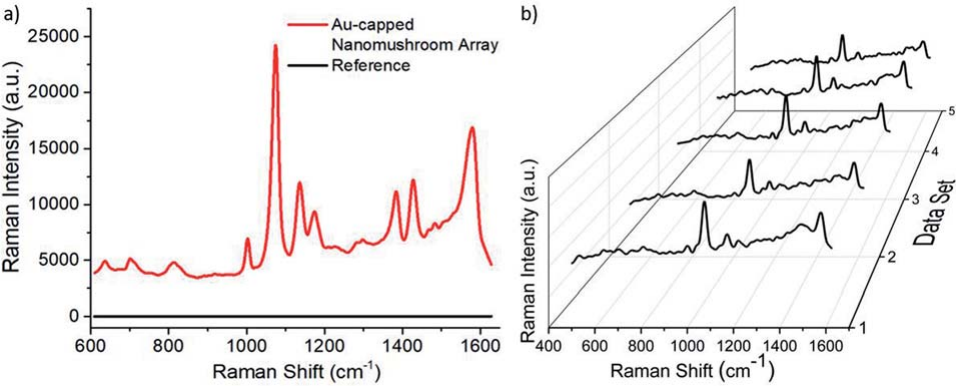
(a) SERS spectrum from 4-ATP adsorbed on the Au-capped mushroom arrays on glass (in red) and on a non-patterned area (in black). (b) 5 measurement points taken from SERS mapping spectra collected with a 5× aperture and 0.5 s acquisition time.

In order to test the effectiveness of the Au-capped nano mushroom arrays as SERS substrates for sensing applications, samples were functionalized with 4-ATP molecules to form a self-assembled monolayer (SAM) through the covalent bond between the gold surface and their thiol group. The size of the nano mushrooms was tuned with the PE-TG process so that the absorption peak maximum matches the laser wavelength at 785 nm.^89^Fig. 7a shows the SERS signals obtained upon laser illumination from a bare area, namely the glass substrate covered with the Au film (in black) and a patterned area (in red). Here, in the case of the Au film no peaks are found since the Raman signal from 4-ATP molecules is too weak to be detected whilst the main fingerprint regions of 4-ATP are clearly visible from the Au-capped nano mushroom arrays and comparable with the ones reported in literature.^90,91^ In particular, the dominant spectral bands peaked at 1074 cm^−1^ and at 1581 cm^−1^, that correspond respectively to the S–C- stretching vibration and to the aromatic ring chain vibrations, confirm the formation of Au–S bond as a result of the SAM formation. The enhancement factor (EF) calculated for the SERS peak at 1074 cm^−1^ is equal to 2.7 × 10^6^ and at 1581 cm^−1^ is 3.3 × 10^6^ (details on the calculation are included in the ESI†). Importantly, the quality of the distribution of the pattern on the substrate, namely the uniformity of the nano mushroom arrays, affects the intensity of the principal SERS peaks. To check this aspect a SERS mapping of the functionalized Au-capped nano mushroom was performed. As shown in Fig. 7b, the SERS mapping is homogeneous confirming that the distribution of the Au-capped nano mushrooms is good enough to make a uniform SERS substrate. A map of 280 × 480 μm^2^ with a step of 15 μm for a total of 660 SERS spectra dataset was collected. The 5 spectra presented represent the four corners and centre of the mapped area. The variance of the SERS peak at 1074 cm^−1^ from the full spectra dataset was calculated from the mapping to be about 9%.

**Fig. 8:**
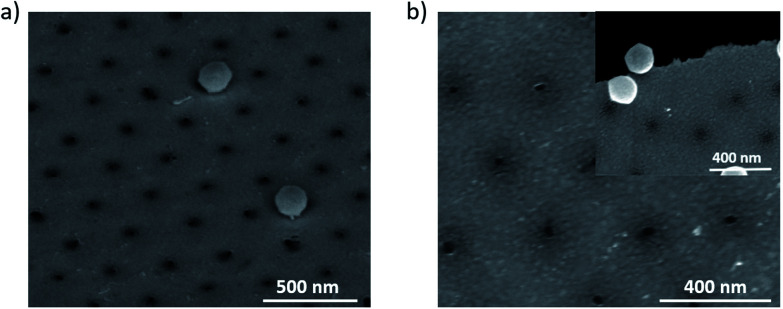
SEM images of nanoapertures array on (a) Si substrate and (b) Si_3_N_4_ membrane, the inset highlights a break to verify the presence of a membrane.

Finally, as a proof of concept to show other capabilities of this mask, ultra-small nanoapertures were produced by using the nano mushrooms pillar as a mask and a sputter deposition of Au with a non-vertical cathode/target orientation, see Fig. 8. For this purpose, it is important to control the Au thickness in order to completely cover the nano mushrooms pillar and the space underneath their hat. We found that the minimum Au thickness to fill the area underneath the mushrooms hat is 30 nm for our deposition conditions, see experimental section. After the removal of the mushrooms we were able to obtain nanoapertures with a mean diameter of 20 nm and standard deviation of 3 nm, as shown in Fig. 8a. The etching process on both Si and Si_3_N_4_ membranes are found to be similar. There are three aspects that should be highlighted: (i) the unique potential of this fabrication route that relies on the PE-TG process to etch the PS bottom part into ultrathin pillars with 20 nm diameter, (ii) the fabrication is cheap as it only relies on a heat source such as an oven/hot plate and an oxygen plasma cleaner both of which are found commonly in laboratories without the necessity for other specialised equipment and (iii) extendable to various substrates. As an example, we produced nanopores on Si_3_N_4_ membranes (see Fig. 8b). The inset of Fig. 8b shows a broken Si_3_N_4_ membrane to firstly show the presence of the nanoapertures on a membrane and an area of bad removal to more easily visually conceptualise the nano mushrooms removed to create the nanoapertures.

## Conclusions

In summary, we have presented a novel class of anisotropic etching processes, termed plasma etching induced by temperature gradients (PE-TG), which we have employed to treat hexagonally close-packed PS sphere arrays on Si/glass substrates and Si_3_N_4_ membranes. We have shown that this etching process is a cost-effective and simple way to shape in 3D colloidal masks from NSL-based fabrication without the use of advanced equipment. We have demonstrated that applying a heat source beneath the substrate plays a crucial role to determine and tune the nano mushrooms' morphology: higher temperature gradient exhibits a faster etching rate at the bottom hemisphere of the PS spheres. Having a nanostructured template, such as the nano mushrooms array, offers the advantage to easily implement fabrication routes to generate plasmonic nanostructures with great potential, such as Au nano mushroom and nanopore arrays. In fact, arrays of Au nano mushrooms are of great interest for biological sensing and the fabrication of cell-electrodes that encourage cellular engulfment behavior,^92,93^ while plasmonic nanopores are an emergent and promising class of biosensor for single molecule detection.^94^ Taking in mind the potential of nanostructured colloidal templates, we believe that PE-TG can be extended to more complex configurations by using different heat sources to generate non-vertical and localized temperature gradients. Furthermore, other plasma etching techniques and conditions (*i.e.* etching species, power, vacuum systems) may be implemented to generalize the PE-TG process to etch different nanomaterials and create other sought-after structures from different lithography-based approaches.

## Experimental section

### Preparation of hcp PS sphere arrays

Negatively charged PS spheres with diameters of 140, 252, 304 and 420 nm monodispersed in 5 wt% aqueous solution were purchased from microParticles GmbH. An interface self-assembly technique was used to create monolayers of hcp PS spheres on glass and silicon (Si) substrates.^95,96^ Briefly, a Si wafer was treated in an oxygen plasma etcher (Gambetti Plasma Cleaner System) for 10 minutes to make the surface hydrophilic and then suspended at an angle of 80° into a glass bath filled with deionized water keeping the bottom edge of the wafer in the water. The stock solution of PS spheres was diluted in 1 : 1 ratio in ethanol and dropped onto the Si wafer. Ordered monolayers of particles were formed as a result of their spreading along the wafer, whilst the excess polystyrene flowed into the water bath. After the suspended wafer has dried naturally with no remaining solvent the Si wafer with the PS monolayers on top was transferred to the air–water interface of a second water bath. The pH of the second deionized water bath was increased to 9 by adding a solution of sodium hydroxide to encourage the compression of the PS monolayers into larger ordered grains. After the transfer was repeated several times to fill the water's surface, an hcp PS sphere monolayer was created. Then, the PS monolayer was ‘fished’ from the water surface onto cleaned Si/glass substrates.

### PE-TG of the hcp monolayer

The hcp PS monolayer on Si/glass was placed on a glass dish preheated in an oven to temperature *T*_glass_ and loaded in the oxygen plasma chamber. The etching power, working distance and oxygen flow rate were fixed at 100 W, 5 cm and 20 sccm respectively. The exposure time was adjusted according to the required features of the nano mushrooms, such as their hat and pillar dimensions.

### Au depositions on PS nano mushrooms

In order to fabricate Au-capped nano mushrooms arrays, an Au layer was deposited using a sputter coater (rate: 22 nm min^−1^, current: 20 mA, Quorum Sputter coater Q150T ES) with a vertical cathode/target orientation. This deposition orientation and the mushrooms' shape prevents the accumulation of Au under their hat, hence only small Au nanoparticles were found on the nano mushrooms' pillar. As a result, the substrate was covered with an Au layer and the nano mushrooms array presented an Au cap with a hole surrounding the bottom part of each pillar.

Alternatively, a Kenosistec KS500 confocal sputter coater with an oblique cathode/target orientation was used to deposit a 30 nm Au layer (rate: 0.047 nm s^−1^, power: 18 W, pressure chamber at high vacuum: *P* < 10^−6^ mbar) on the PS nano mushrooms array, filling also the space under their hat. After the removal of the mushrooms with tape, an ordered array of nanoapertures with a size of approximately 20 nm was obtained. The entire process was repeated on 100 nm thick silicon nitride (Si_3_N_4_) membranes to show that this process can be adopted to fabricate nanopore arrays.

### Optical and morphological characterization

A focused ion beam scanning electron microscope (FEI Helios NanoLab 650) was used to obtain the SEM images. The absorption spectra were recorded with a Cary 5000 spectrophotometer operated at a resolution of 1 nm in the wavelength range between 300 and 1000 nm. The polarized transmittance and reflectance spectra at multiple angles of incidence from 0° to 40° were acquired in the spectral range 300–1500 nm with a J. A. Woolam Co. V-VASE ellipsometer.

### Functionalization with 4-amino-thiophenol (4-ATP)

A solution of 4-ATP (concentration 10^−4^ M in ethanol) was pipetted on top of the Au-coated colloidal monolayer. After 9 hours of incubation a SAM of 4-ATP molecules was formed and the excess was removed washing the sample several times with ethanol. Finally, the sample was dried with a nitrogen gun.

### Raman measurements

A Renishaw inVia Raman spectrometer equipped with a 20× objective and a 785 nm laser was used to perform Raman measurements. The laser beam was adjusted to have a spot size of 9.2 μm, power 10% and an exposure time of 0.1 s unless specified elsewhere.

## Conflicts of interest

There are no conflicts to declare.

## Supplementary Material

NA-003-D0NA00718H-s001
