# Supplementary material for: Breaking the symmetry of nanosphere lithography with anisotropic plasma etching induced by temperature gradients
Source: Nanoscale Adv. 2020 Dec 11;3(2):359–69. doi: 10.1039/d0na00718h (PMC9419189; doi:10.1039/d0na00718h)
Supplement: NA-003-D0NA00718H-s001 [file NA-003-D0NA00718H-s001.pdf]

Electronic Supplementary Material (ESI) for Nanoscale Advances.
This journal is © The Royal Society of Chemistry 2020

Supporting Information

Breaking the Symmetry of Nanosphere Lithography with Anisotropic Plasma Etching Induced by Temperature Gradients

Daniel Darvill, [†a] *Marzia Iarossi,* [†ab] *Ricardo M. Abraham Ekeroth,*[ac] *Aliaksandr Hubarevich,* [a] *Jian-An Huang,* [a] *and Francesco De Angelis*[*a]

[a]Istituto Italiano di Tecnologia, Via Morego 30, 16136 Genova, Italy

[b]Dipartimento di Informatica, Bioingegneria, Robotica e Ingegneria dei Sistemi (DIBRIS). Università degli Studi di Genova, Via Balbi 5, 16126 Genova, Italy

[c]Instituto de Física Arroyo Seco (CIFICEN-CICPBA-CONICET), Universidad Nacional del Centro de la Provincia de Buenos Aires, Pinto 399, 7000 Tandil, Argentina

*Email: francesco.deangelis@iit.it

Morphological characterization of hcp PS sphere arrays treated with oxygen plasma etching and in contact with a glass dish at room temperature i.e. no temperature gradient applied.

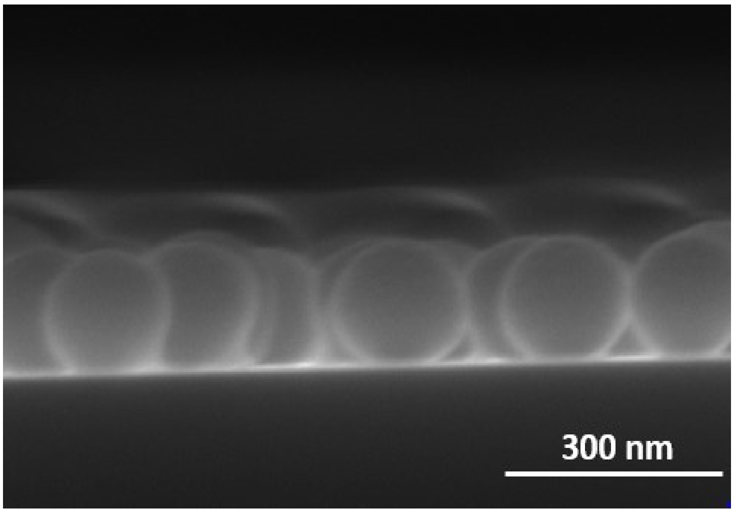

Figure S1. Cross-section SEM image of hcp PS sphere arrays (initial particles diameter 304 ± 10 nm) treated with an oxygen plasma etching process (power: 100 W, working distance: 5 cm, oxygen flow rate: 20 sccm) for 120s. The sample is placed on a glass dish at room temperature.

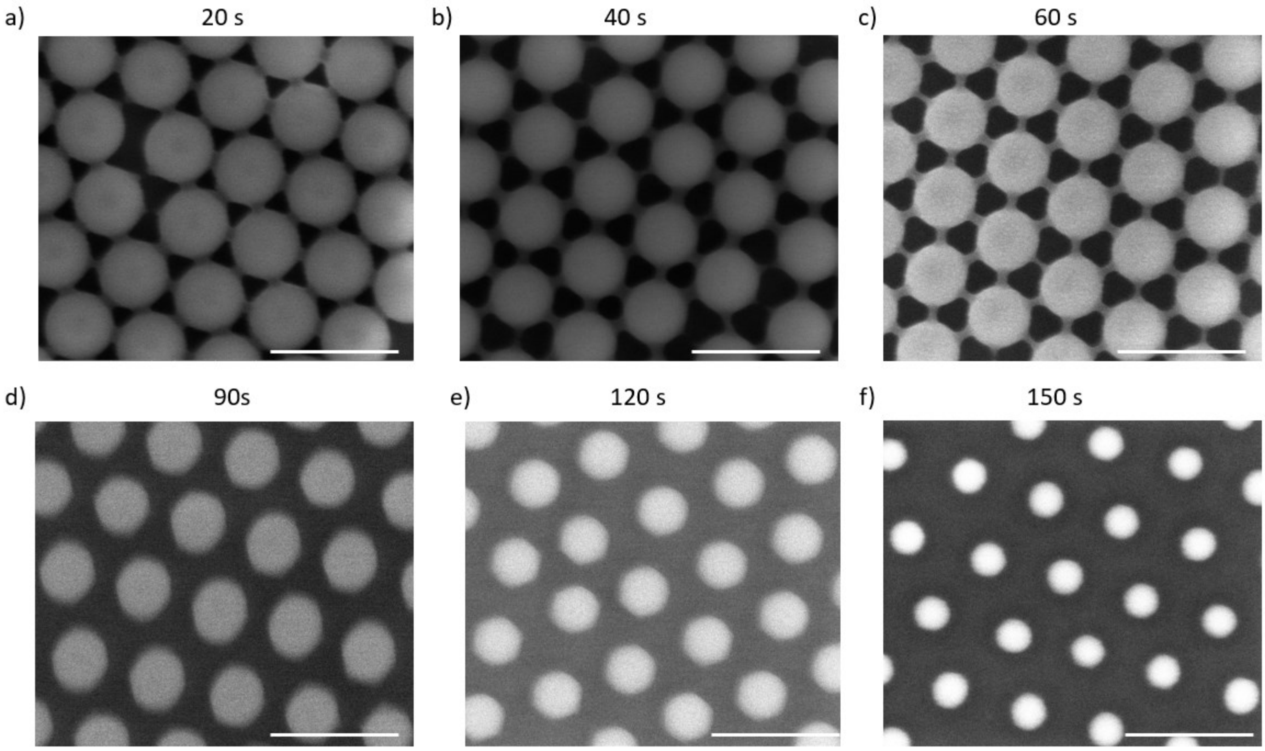

Figure S2. (a-f) SEM images (scale bar: 500 nm) of hcp PS sphere arrays (initial particles diameter 304 ± 10 nm) treated with an oxygen plasma etching process (power: 100 W, working distance: 5 cm, oxygen flow rate: 20 sccm) at various exposure times.

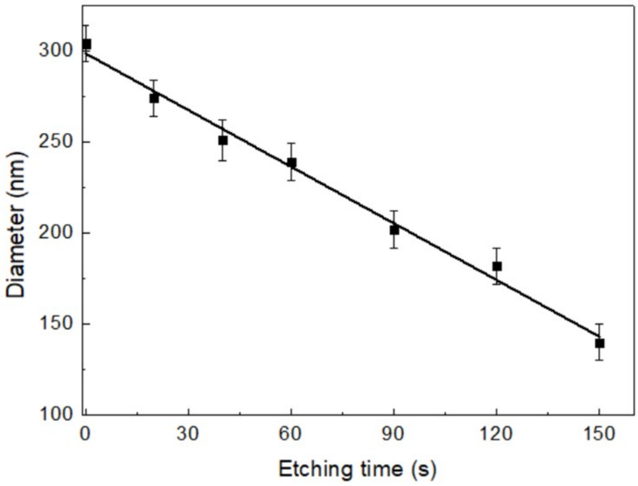

Figure S3. Average diameter of the PS spheres after the etching process as a function of the exposure time.

Comparison between the PE-TG process of isolated spheres and grains of particles.

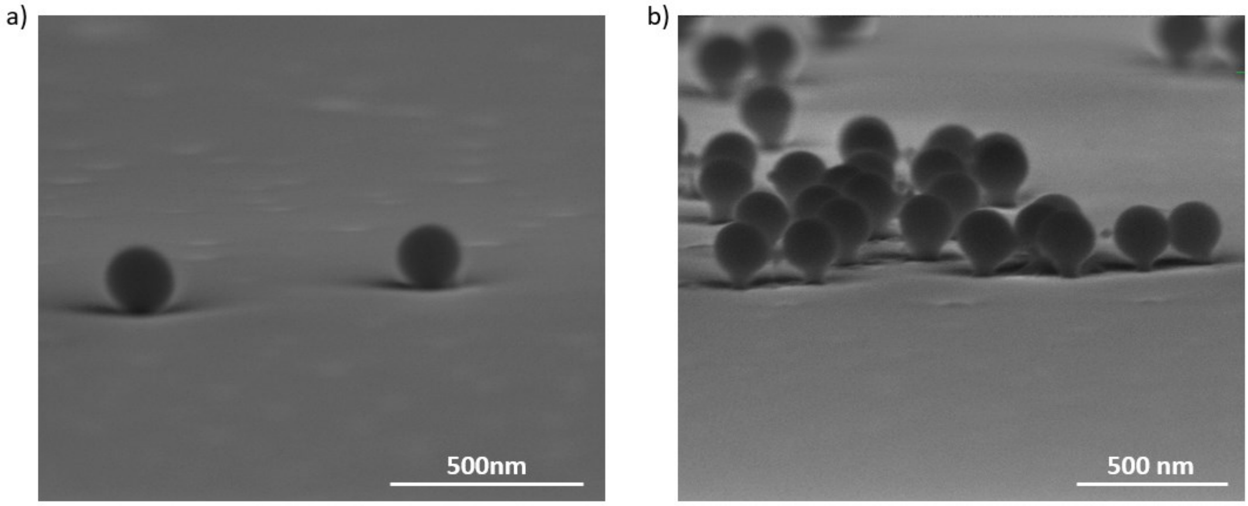

Figure S4. Cross-section SEM images of non-ordered PS spheres arrays after PE-TG process for 120s at 65 °C. In (a) isolated particles are still spherical after the etching process. In (b) mushrooms shaped PS particles are obtained in areas where the spheres were in contact with each other.

Thermodynamic simulations.

Laminar Flow of air at low pressure as well as heat transfer physics were considered in the frame of a Finite Element Solver. Hexagonally close-packed arrays of PS spheres were simulated by considering periodic boundary conditions along the vertical surfaces of a hexagonal prism that corresponds to a Wigner-Seitz cell. In the case of single-sphere simulations, these boundary conditions were replaced by conditions representing open boundaries. In order to generate a temperature gradient on the nanometer scale and explain the anisotropic etching observed on the hcp PS array it is essential to include the air flow in the simulations. For the heat-transfer physics the temperature of the glass dish T_{glass} used in the experiments was set as the bottom surface of the simulation prism and kept constant whilst the initial value of the temperature of the environment started at room temperature. The sphere surface as well as the substrate surface were considered as diffuse surfaces that radiate heat to complete the description of the thermal simulations. However, the radiative emission was found not to give a significant contribution to the final temperatures. The temperature gradient across the system is obtained in a few microseconds for all cases. Thus, the time required to build the temperature gradients is negligible with respect to the etching times considered in this work.

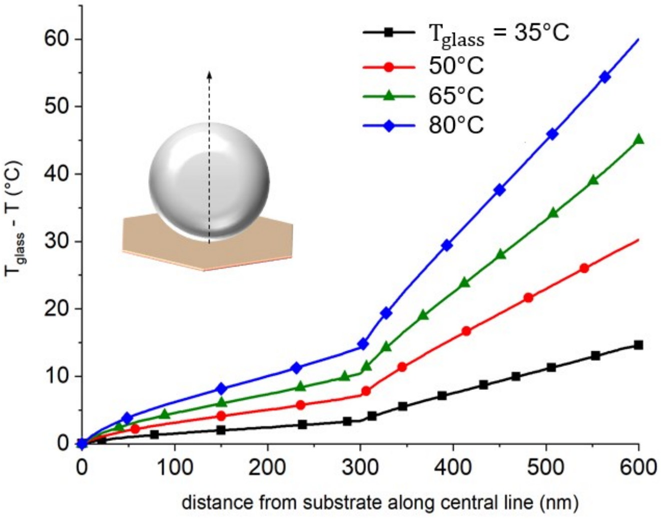

Figure S5. Profiles of the temperature difference T_{glass} -T along a central line (inset scheme) as a function of the distance from the substrate for a hcp array of PS spheres with a diameter of 300 nm on glass.

An estimation of the anisotropic etching induced by PE-TG as a function of the substrate temperature can be found in Figure S5. This figure shows the profiles of the temperature difference T_{glass} -T as a function of the distance from the substrate along a central line (see inset scheme). Each line corresponds to a variation of the substrate temperature T_{glass}. Note how the lines increase their initial slope when T_{glass} is increased from 35 °C up to 80 °C. There is a significant temperature increase at 300 nm distance from the substrate because the material's properties change from PS to air, and thus the temperature difference reaches the value T_{glass}-T_0, where T_0 = 20 °C is room temperature. Thus, PE-TG process becomes more anisotropic when T_{glass} is increased. In these conditions the heat flow is accumulated at the bottom hemisphere of the particles creating an anisotropic etching rates.

Further optical characterization of Au mushroom arrays.

The optical properties of the Au mushroom arrays can be tuned by varying the gap (H_G) or the diameter of their hat (D_H), namely by adjusting the etching parameters during the PE-TG process. It is worth noticing that the value of gap (H_G) is determined by the pillar's height and the Au thickness (Figure S7a), and thus it can be tuned by applying a different temperature during the PE-TG treatment

or changing the thickness of the Au layer. In order to highlight how the nanostructures change after the deposition of Au, two cross-sections SEM images of nano mushrooms after the deposition of 10 and 20 nm of Au are reported in Figure S6a-b respectively. By increasing the layer thickness the reduction of the gap as well as the increase of the Au cap size are well-visible.

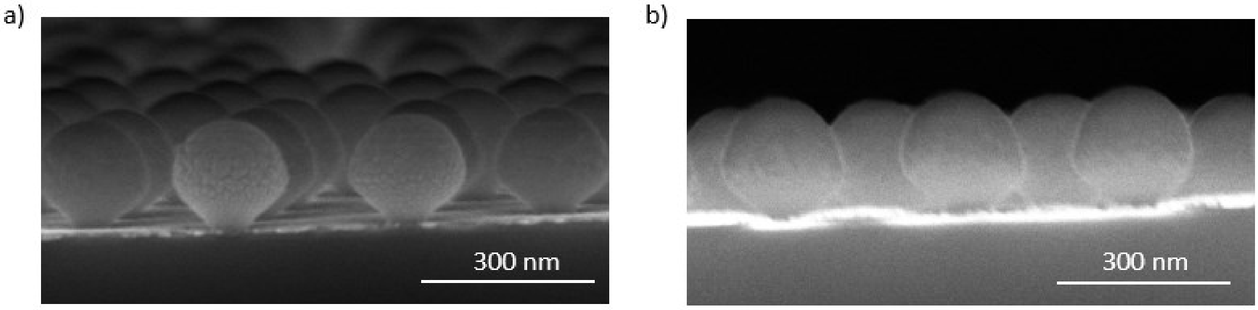

Figure S6. Cross section SEM images of Au-capped mushroom arrays: (a) and (b) the thickness of the Au layer is 10 and 20 nm respectively.

The diameter of the mushrooms hat is controlled by the exposure time of the etching process, in a way that smaller hats are obtained for a longer exposure. Figure S7b shows the transmission spectra of Au nano mushrooms array as a function of D_H and fixing the thickness of the Au layer at 30 nm. A red-shift of the second transmission peak from 940 to 1360 nm has been observed increasing D_H from 85 to 250 nm. This result is expected because by fixing the cap thickness a larger cap size has a plasmonic resonance centered at longer wavelengths, due to their larger aspect ratio.

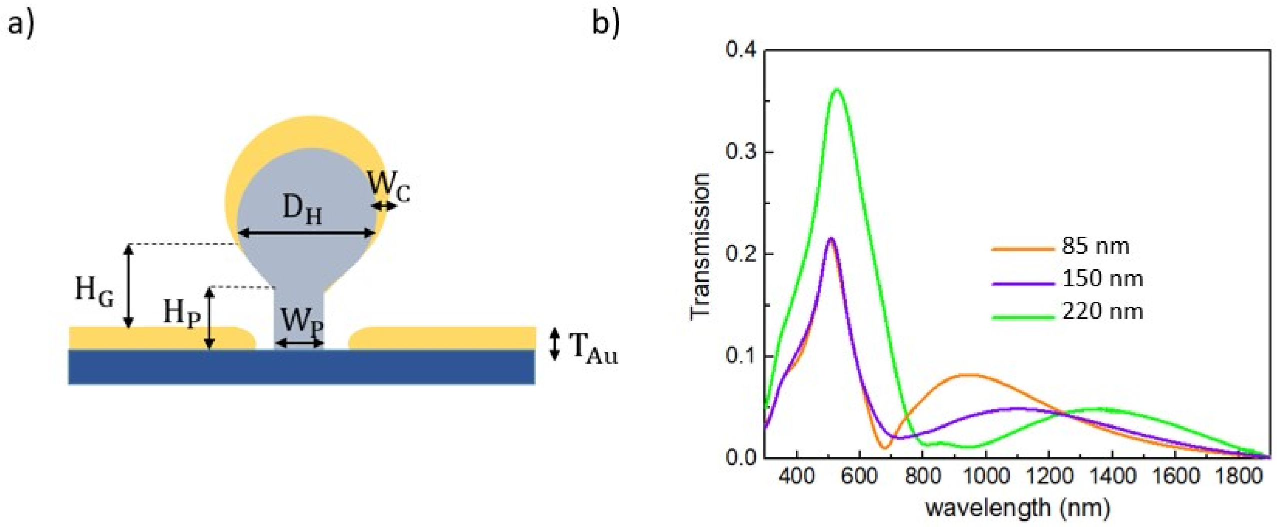

Figure S7. (a) Scheme of the principal features that affect the optical properties of Au-capped mushroom arrays. (b) Transmission spectra of Au-capped mushroom arrays at various $D_{H.}$

Finally, the polarization and angular dependence of the Au-capped mushroom arrays has been studied acquiring transmittance and reflectance spectra for p- an s- polarizations at various angles (Figure S8a and b). The spectra are independent from light polarization, whilst a red-shift of about 40 nm has been observed changing the incidence angle from 20° to 40°. These measurements refer to Au-capped mushrooms arrays obtained from 300 nm hcp PS sphere arrays treated with PE-TG at 65 °C for 120 s and covered with 30 nm layer of Au. As estimated from SEM images, D_H and H_P are about 150 and 45 nm, respectively (Figure S8c).

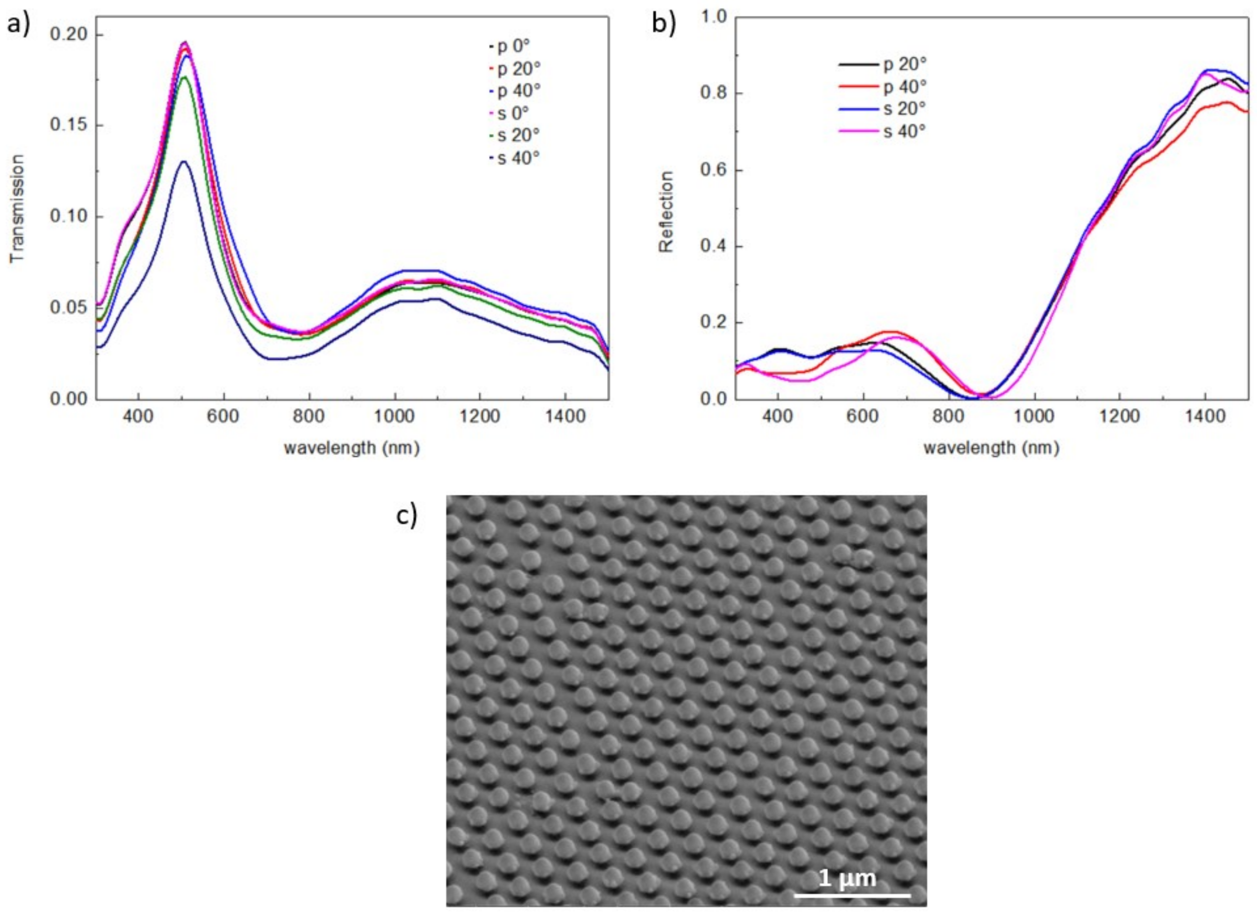

Figure S8. (a) Transmittance and (b) reflectance spectra of Au-capped nano mushrooms arrays for p- and s- polarization at various angles of incidence. (c) Tilted-view SEM image of Au-capped mushroom arrays.

Calculation of the enhancement factor.

The Enhancement Factor (EF) of the Au-capped mushroom arrays substrate has been calculated from the following equation:

$$EF = \frac{I_{SERS}}{I_{RS}} \cdot \frac{C_{RS} H_{eff}}{\mu_M \mu_S A_M}, \quad (1)$$

where I_{SERS} and I_{RS} are the Raman intensities of the 4-ATP molecules under SERS and non-SERS respectively, C_{RS} [μm^{-3}] is the volume density of the molecules used for the non-SERS measurement, H_{eff} [µm] is the effective height of the scattering volume from the objective, μ_M [μm^{-2}] is the surface

density of the single nanostructures producing the enhancement (in our case the Au film plus the nano mushroom's caps), μ_S [μm^{-2}] is the surface density of the 4-ATP on the Au and A_M [μm^2] is the surface area of the Au layer plus one nano mushroom's cap. In order to calculate the quantities in equation (1) related to the non-SERS measurements, the Raman spectrum of 4-ATP solid films of known volume and weight on a glass slide was measured. From our Raman set-up with a 20x objective the measured H_{eff} is 350 μm and the calculated C_{RS} of the 4-ATP film is 4.94 × 109 μm^{-3}. Moreover, taking into account the SEM images of the SERS substrate we estimated that μ_S is equal to 3.3 · 106 molecules/μm^2, μ_M is about 6 μm^{-2}. The intensities at 1074 cm^{-1} for SERS and non-SERS measurements are 24710 and 560 respectively, at 1581 cm^{-1} are 16815 and 315 respectively. With this information the EFs reported in the Results and Discussion of the manuscript were calculated.
